# Comparison of two-year outcomes after photodynamic therapy with ranibizumab or aflibercept for polypoidal choroidal vasculopathy

**DOI:** 10.1038/s41598-017-16476-1

**Published:** 2017-11-28

**Authors:** Wataru Kikushima, Yoichi Sakurada, Atsushi Sugiyama, Seigo Yoneyama, Naohiko Tanabe, Mio Matsubara, Fumihiko Mabuchi, Hiroyuki Iijima

**Affiliations:** Departments of Ophthalmology, Yamanashi University, Yamanashi, Japan

## Abstract

Photodynamic therapy (PDT) combined with intravitreal anti-vascular endothelial growth factor (VEGF) agents is currently the first-line treatment for polypoidal choroidal vasculopathy (PCV), along with anti-VEGF monotherapy. In this study, 100 eyes with treatment-naïve PCV were initially treated with PDT combined with intravitreal ranibizumab (IVR; n = 57) or aflibercept (IVA; n = 43). We compared two-year outcomes between these two groups and investigated factors associated with visual improvement and retreatment over 24 months. Best-corrected visual acuity (BCVA) was significantly improved in both groups (P < 0.001) at 24 months. Multiple regression analysis revealed that visual improvement at 24 months was associated with female (P = 0.030), worse baseline BCVA (P = 3.0 × 10^−6^), smaller greatest linear dimension (GLD; P = 2.0 × 10^−4^), and treatment with IVA rather than IVR (P = 0.016). Multiple logistic regression analysis revealed that absence of retreatment was associated with younger age (P = 2.2 × 10^−4^), female (P = 1.2 × 10^−3^), and the non-risk variants of *ARMS2* A69S (P = 6.0 × 10^−4^). Although there were no significant differences in the retreatment rate between the two groups, PDT/IVA may be superior to PDT/IVR in terms of visual improvement at 24 months.

## Introduction

Polypoidal choroidal vasculopathy (PCV), which is characterized by polypoidal dilation with or without branching vascular networks on indocyanine green angiography (ICGA)^1^, exhibits recurrent serosanguineous detachment of the sensory retina and/or retinal pigment epithelium^2^. PCV is more prevalent in Asians than in Caucasians^3–8^, and a few clinic-based studies in Japan have demonstrated that PCV accounts for almost half of the eyes with age-related macular degeneration (AMD)^7,8^.

Among several genetic variants associated with AMD, it has been reported that *CFH* and *ARMS2* variants are strongly associated with AMD including PCV^9–11^. Moreover, these variants are reportedly associated with clinical phenotype in PCV^12–14^.

Photodynamic therapy (PDT) with verteporfin was the first approved treatment option for exudative AMD, including PCV. However, eyes with PCV often develop hemorrhagic complications after PDT monotherapy^15^.

PDT theoretically causes photochemical thrombotic occlusion of polypoidal vascular lesions^16^, while also inducing the upregulation of VEGF as an adverse side effect. Intravitreal injection of anti-VEGF agents is expected to decrease the high intraocular concentration of VEGF, which causes exudation of both intraretinal and subretinal fluid. Combination therapy with PDT and intravitreal anti-VEGF agents has been demonstrated to reduce the incidence of PDT-related hemorrhagic complications relative to treatment with PDT alone^17^, and is also more effective for improving visual acuity^18^ and reducing retreatment than intravitreal anti-VEGF-agent monotherapy in eyes with PCV^19,20^.

Several studies have reported two-year results with favorable visual outcomes for PDT combined with intravitreal ranibizumab (IVR) in eyes with PCV^21–23^. Another anti-VEGF agent, aflibercept, is a recombinant fusion protein that binds to VEGF-A, VEGF-B, and placental growth factors with a higher binding affinity than other available anti-VEGF agents, including ranibizumab and bevacizumab^24^. The one-year results that have been reported for PDT combined with intravitreal aflibercept (IVA) are favorable^19,20,25^.

In the present study, we compared the two-year results of visual outcome and retreatment between eyes with PCV treated with PDT and either IVR or IVA.

## Results

**Figure 1:**
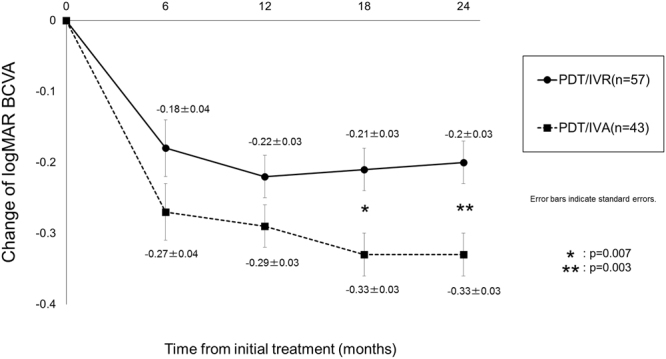
Changes of best-corrected visual acuity in eyes with polypoidal choroidal vasculopathy treated with photodynamic therapy with intravitreal ranibizumab or aflibercept.

There were no significant differences in the genetic or clinical characteristics of the patients between the two treatment groups (Table 1). Compared with baseline BCVA, a significant improvement in BCVA was seen at all points in both groups (P < 0.001; Fig. 1). Although there were no significant differences in visual improvement between the two groups at 6 or 12 months, it was significantly greater in the PDT/IVA group at 18 and 24 months (P = 0.007 and 0.003, respectively). There was a significant difference in BCVA between the 2 groups at 18 and 24 months (P = 0.01 and 0.009, respectively).

**Table 1:** Clinical and genetic characteristics of the patients according to treatment modality.

	All patients (n = 100)	PDT/IVR group (n = 57)	PDT/IVA group (n = 43)	P value
Age	72.9 ± 8.6	72.4 ± 8.6	73.6 ± 8.7	0.71
Male gender	70(70.0%)	42 (73.7%)	28 (65.1%)	0.35
Current smoker	14(14.0%)	9(15.8%)	5(11.6%)	0.55
Baseline logMAR BCVA	0.54 ± 0.28	0.55 ± 0.27	0.52 ± 0.29	0.52
Greatest linear dimension (μm)	1844 ± 874	1957 ± 942	1694 ± 760	0.25
Central macular thickness (µm)	388 ± 111	393 ± 119	381 ± 99	0.74
***ARMS2*** **A69S (rs10490924)**				
TT	36(36.0%)	23(40.4%)	13(30.2%)	
TG	44(44.0%)	25(43.9%)	19(44.2%)	
GG	20(20.0%)	9(15.7%)	11(25.6%)	
T-allele frequency	58.0%	62.3%	52.3%	0.16
***CFH*** **I62V (rs800292)**				
GG	53(53.0%)	27(47.4%)	26(60.4%)	
GA	41(41.0%)	25(43.9%)	16(37.2%)	
AA	6(6.0%)	5(8.8%)	1(2.3%)	
G-allele frequency	73.5%	69.3%	79.1%	0.12

ARMS: age-related maculopathy susceptibility, BCVA: best-corrected visual acuity.

CFH: complement factor H, logMAR: logarithm of the minimal angle resolution.

A better BCVA (or lower logMAR BCVA) at 24 months was significantly associated with female sex, better baseline BCVA, PDT/IVA treatment, and smaller GLD (Table 2). A greater increase in BCVA (or decrease in logMAR BCVA) at 24 months was significantly associated with female sex, worse baseline BCVA, PDT/IVA treatment, and smaller GLD.

**Table 2:** Baseline factors associated with BCVA(logMAR) and visual gain at 24 months.

Variables	BCVA at 24 months		Visual gain at 24 months	
	β-coefficient	p-value	β-coefficient	p-value
Age	0.136	0.055	0.179	0.055
Male gender	0.16	0.030	0.21	0.030
Current smoker	0.057	0.41	0.075	0.41
Baseline BCVA (logMAR)	0.53	2.2 × 10^−10^	−0.49	3.0 × 10^−6^
Treatment group (PDT/IVR:0, PDT/IVA:1)	−0.17	0.016	−0.22	0.016
Central macular thickness (μm)	−0.078	0.25	−0.10	0.25
Greatest linear dimension (μm)	0.225	2.0 × 10^−4^	0.295	2.0 × 10^−4^
*ARMS2* A69S T allele	0.055	0.42	0.072	0.42
*CFH* I62V G allele	−0.043	0.53	−0.057	0.53

BCVA: best-corrected visual acuity, LogMAR: logarithm minimal angle of resolution, AMD: age-related macular degeneration, IVR: intravitreal ranibizumab, IVA: intravitreal aflibercept injection, PCV: polypoidal choroidal vasculopathy, *ARMS*: age-related maculopathy susceptibility, *CFH*: complement factor H.

During the 24-month period, 17 of the 43 (39.5%) PDT/IVA patients and 25 of the 57 patients (43.8%) PDT/IVR patients required no retreatment. The mean number of additional PDT/IVA or PDT/IVR treatments was 0.16 ± 0.37 and 0.26 ± 0.53, respectively, which was not significantly different (P = 0.35). The mean number of additional IVA or IVR treatments without PDT was 1.6 ± 1.7 and 1.8 ± 1.6, respectively, which was also not significantly different (P = 0.57).

**Figure 2:**
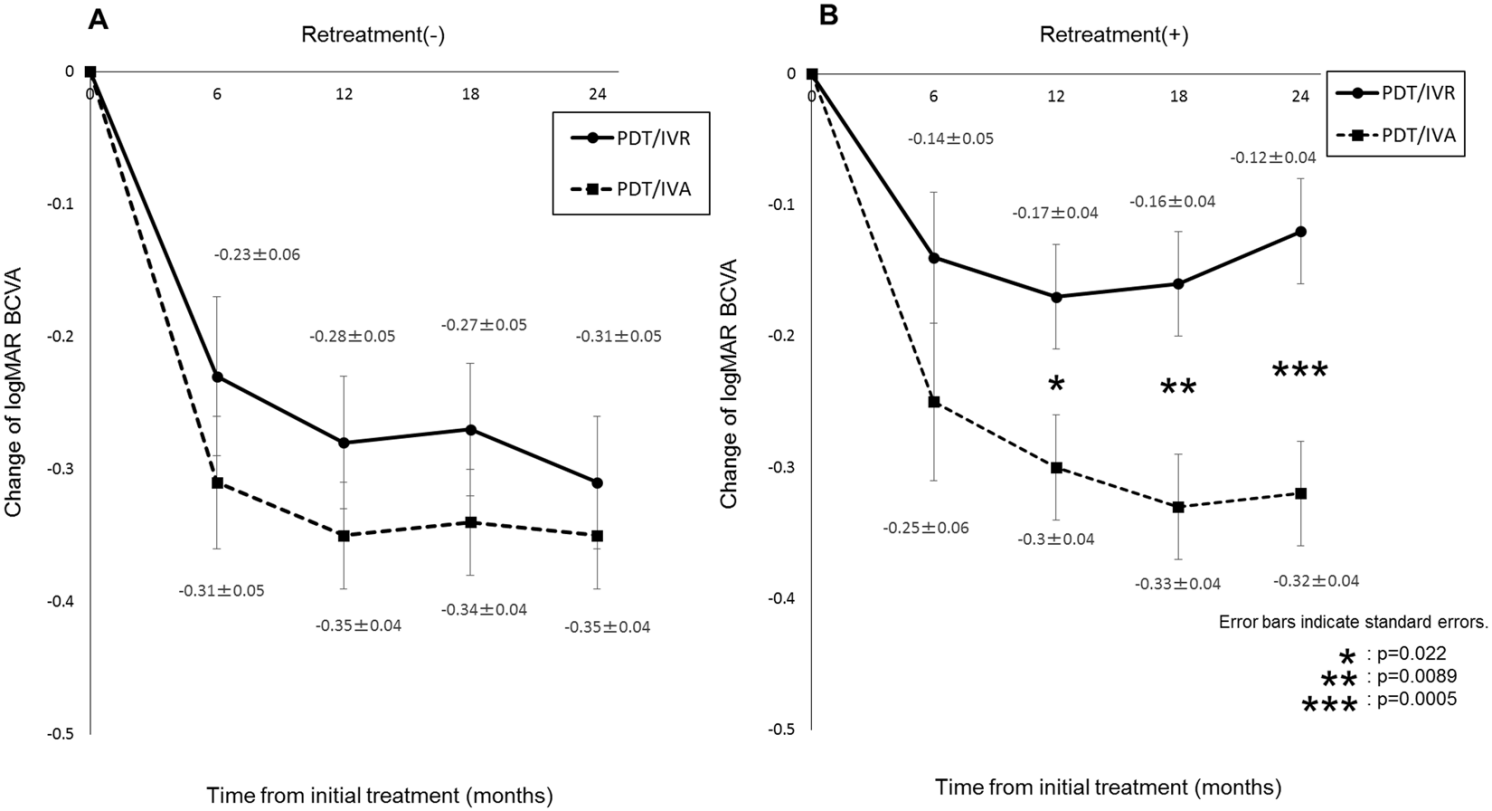
Changes of best-corrected visual acuity in eyes with polypoidal choroidal vasculopathy according to the requirement of retreatment.

**Figure 3:**
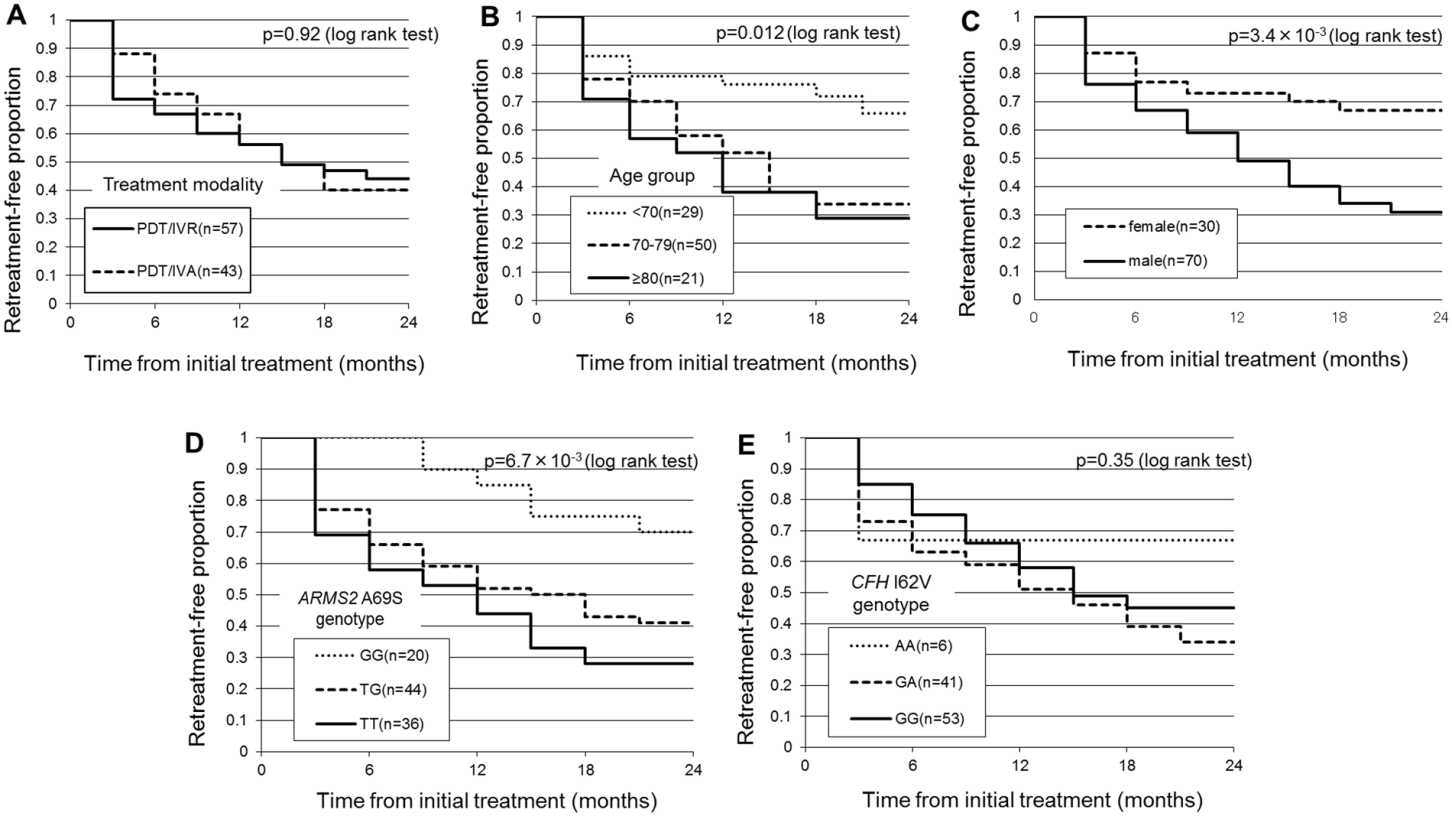
Kaplan-Meier plots showing retreatment-free proportion by treatment modality (**A**), age groups (**B**), gender (**C**), ARMS2 A69S genotypes (**D**) and CFH I62V genotypes.

**Figure 4:**
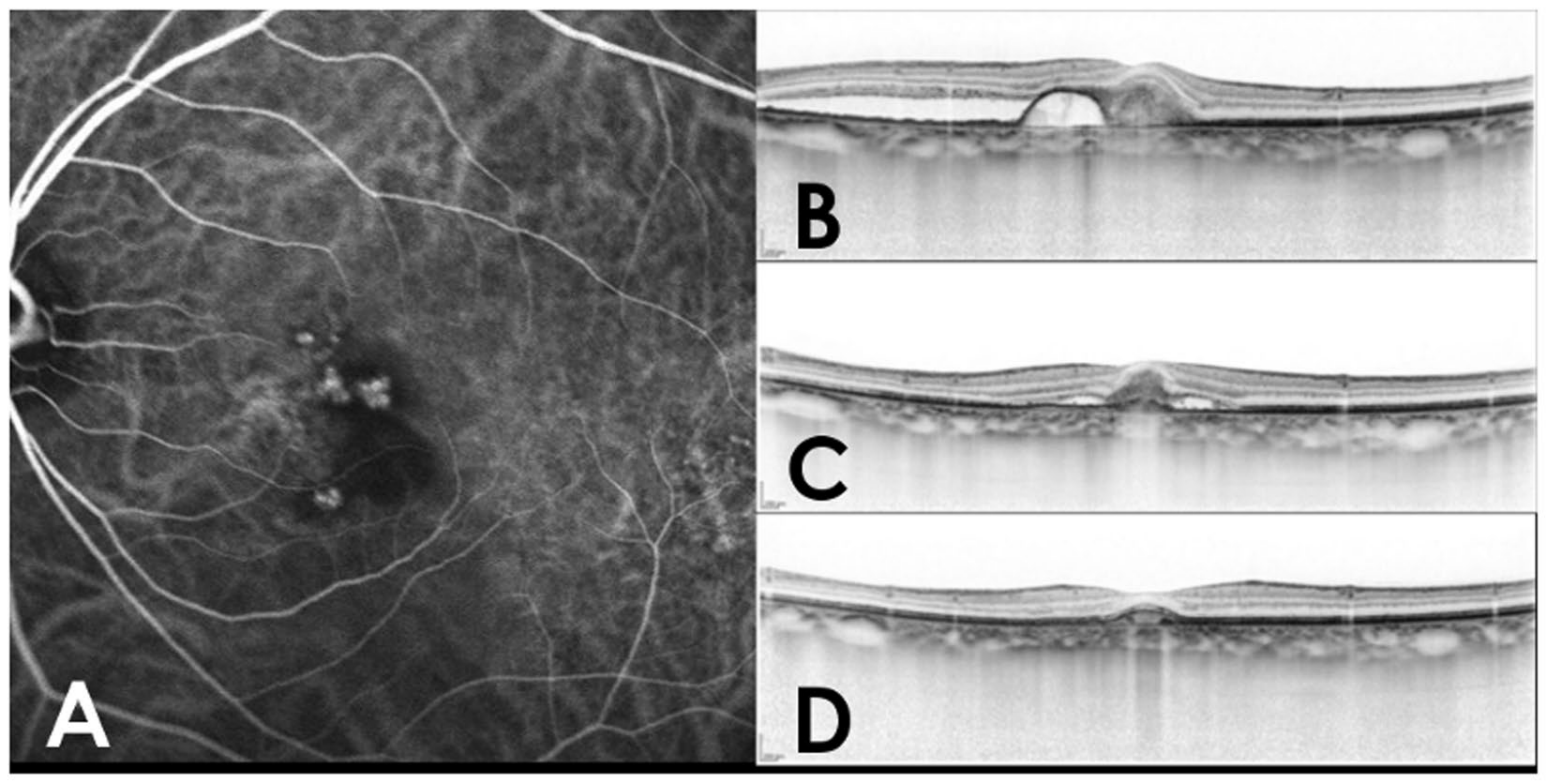
A 60-year old male patient with polypoidal choroidal vasculopathy treated with involving photodynamic therapy and intravitreal aflibercept injection in the left eye. (**A**) Multiple polypoidal lesions with branching vascular network were found on the fovea on indocyanine green angiography. (**B**) A vertical OCT scan showed serous pigmental epithelial detachment and subretinal fluid on the initial visit. His visual acuity was 0.4 in the left eye. (**C**) A vertical OCT scan 6 months after the initial combination therapy showed recurrence of subretinal fluid in spite of dry macula at 3 months. An additional intravitreal injection of aflibercept was administrated. (**D**) A vertical OCT scan 2-year after the initial combination therapy showed no exudation. The visual acuity improved to 1.0 in the left eye at 24 months.

Although visual improvement was greater in the PDT/IVA group than in the PDT/IVR group in eyes without retreatment, the difference was not significant at any point during 24 months of follow-up (Fig. 2). In retreated eyes, however, improvement in BCVA was significantly greater in the PDT/IVA group than in the PDT/IVR group at 12, 18, and 24 months (P = 0.022, 0.0089, and 0.0005, respectively). According to both logistic regression testing and the Cox proportional hazards regression model, the necessity of the retreatment was associated with older age, male sex, the *ARMS2* A69S risk variants (T allele, Tables 3 and 4). Kaplan-Maier survival analysis confirmed this: younger age, female sex, and non-risk alleles for *ARMS2* A69S were associated with a lower risk of retreatment (Fig. 3). A representative case requiring retreatment is shown in Fig. 4.

**Table 3:** Clinical and genetic factors associated with retreatment after initial treatment.

	Without retreatment (n = 42)	With retreatment (n = 58)	Univariate p-value	Multivariate p-value	Odds ratio	95% confidence interval
Age	69.2 ± 9.5	75.5 ± 6.8	3.2 × 10^−4^	2.2 × 10^−4^	1.16	1.07–1.25
Male gender	22(52.4%)	48(82.8%)	1.1 × 10^−3^	1.2 × 10^−3^	8.15	2.29–29.1
Current smoker	5(11.9%)	9(15.5%)	0.61	0.12	3.45	0.73–16.3
Treatment modality (0: PDT/IVR, 1: PDT/IVA)	17(40.5%) (25:17)	26(44.8%) (32:26)	0.66	0.26	1.86	0.64–5.44
Baseline log MAR BCVA	0.50 ± 0.28	0.56 ± 0.27	0.22	0.38	0.42	0.058–2.97
Greatest linear dimension (μm)	1827 ± 899	1856 ± 863	0.86	0.98	1.0	1.0–1.0
Central macular thickness (µm)	385 ± 92	391 ± 123	0.59	0.79	1.0	1.0–1.0
*ARMS2* A69S (rs10490924) T-allele frequency (TT: TG: GG)	45.2% (10:18:14)	67.2% (26:26:6)	1.9 × 10^−3^	6.0 × 10^−4^	4.59	1.92–11.0
*CFH* I62V (rs800292) G-allele frequency (GG: GA: AA)	73.8% (24:14:4)	73.3% (29:27:2)	0.93	0.95	0.97	0.40–2.38

BCVA: best-corrected visual acuity, *ARMS*: age-related maculopathy susceptibility, *CFH*: complement factor H, log MAR: logarithm of the minimal angle resolution, IVR: intravitreal ranibizumab injection, IVA: intravitreal aflibercept injection, PDT: photodynamic therapy.

## Discussion

In the present study, we compared the two-year outcomes of visual improvement and retreatment in eyes with PCV receiving initial PDT/IVR or PDT/IVA treatment. While BCVA significantly improved at 24 months in both groups, the amplitude of improvement was significantly greater in the PDT/IVA group (Fig. 1). This difference was mainly due to the subset of eyes requiring retreatment because of the recurrence of exudation (Fig. 2).

Because aflibercept has a longer half-life in the vitreous than ranibizumab^26^, it may more effectively resolve recurring exudation, leading to the observed greater improvements in visual gain. Several studies have reported that switching from ranibizumab to aflibercept is effective in resolving persistent exudative changes in eyes with PCV refractory to ranibizumab^27,28^. Therefore, an improvement in logMAR BCVA (of up to 0.3 units) in eyes requiring additional retreatment in the PDT/IVR group could presumably have been achieved and maintained if they had then been treated with IVA instead of IVR. The smaller improvement in BCVA for all subjects in the PDT/IVR group may have resulted from IVR being less effective in absorbing exudation in the eyes that required retreatment.

The greater improvement in BCVA in eyes with a poorer baseline BCVA (Table 2) could be explained by the ceiling effect, in which eyes with the poorer baseline BCVA have a larger range for improvement than those with a better baseline BCVA. Regarding the association between a smaller GLD and greater improvements in BCVA (Table 2), Tsujikawa *et al*.^29^ reported that in eyes with a smaller lesion size (less than one-disc diameter) on ICGA, which may be equivalent to GLD, during at least 24 months of follow up, the visual prognosis was better than in those with a larger lesion size. Several other reports have also demonstrated that lesion size is a prognostic factor in eyes with PCV after PDT, with or without anti-VEGF agents^30,31^. The greater improvement in BCVA at 24 months in females (Table 2) may be related to the fact that females experienced less recurrence (Table 4).

**Table 4:** Factors associated with retreatment-free period after combination therapy (Cox-regression analysis).

	β-coefficient	p-value	Hazard ratio	95% confidence interval
Age	0.072	2.8 × 10^−4^	1.07	1.03–1.11
Male gender	1.07	5.5 × 10^−3^	2.92	1.37–6.24
Current smoker	0.71	0.060	2.03	0.97–4.24
Treatment modality (0: IVR/PDT, 1: IVA/PDT)	0.11	0.70	1.12	0.64–1.95
Baseline log MAR BCVA	−0.87	0.11	0.42	0.14–1.22
Greatest linear dimension (μm)	−9.6 × 10^−5^	0.11	0.42	0.14–1.22
Central macular thickness (µm)	−3.8 × 10^−4^	0.78	1.0	1.0–1.0
*ARMS2*A69S (rs10490924) T-allele	0.66	1.3 × 10^−3^	1.94	1.37–6.24
*CFH*I62V (rs800292) G-allele	−0.22	0.33	0.80	0.51–1.26

BCVA: best-corrected visual acuity, *ARMS*: age-related maculopathy susceptibility, *CFH*: complement factor H, log MAR: logarithm of the minimal angle resolution, IVR: intravitreal ranibizumab injection, IVA: intravitreal aflibercept, PDT: photodynamic therapy.

With respect to our finding that retreatment was associated with older age, male sex, and the risk variants of *ARMS2* A69S, irrespective of treatment type (Fig. 3, Table 4), we have recently reported that two of these factors—older age and the risk variants of *ARMS2* A69S—are associated with retreatment due to residual or recurrent exudation after three-monthly IVA for exudative AMD^23^. The present results indicate the same tendency. The reason for the association with the male sex is unclear, and was also not understood in the previous study.

There are several limitations in the present study. The first is the retrospective nature of the analysis. The second is that the resolution of the polypoidal lesions could not be consistently investigated, because the FA/ICGA examination was conducted only at baseline, three months after the initial therapy, and at the time of recurrence. The third is the absence of the data on choroidal thickness in this study, which came about because not all eyes were examined by EDI-OCT. Recently, subfoveal choroidal thickness was shown to be an important prognostic factor for AMD, including PCV^32,33^. Further studies are therefore necessary to determine whether choroidal thickness responds to PDT/IVR or PDT/IVA treatment. In the present study, the interval between IVR or IVA treatment and PDT was seven days. Sato *et al*.^34^ reported that a two-day interval resulted in better visual outcomes in eyes with PCV treated with PDT/IVR; however, this result was found to be controversial in a systematic review and meta-analysis of comparative studies^35^. Further analyses are thus required to determine the optimal interval between the intravitreal injection of anti-VEGF agents and PDT.

In conclusion, the necessity of retreatment for PCV after initial PDT in combination with either IVR or IVA therapy is associated with older age, male sex, and the risk variants of *ARMS2* A69S. Although there was no significant difference in the rate of retreatment between the therapeutic approaches during the 24-month follow-up period, PDT/IVA may be superior to PDT/IVR in terms of visual improvement.

## Methods

### Subjects

We retrospectively reviewed the medical charts of 100 consecutive eyes from 100 patients with treatment-naïve PCV who received PDT combined with either IVR (n = 57) or IVA (n = 43) and underwent follow-up for at least 24 months. The choice of PDT/IVR or PDT/IVA treatment depended on when they were treated at our clinic; the two treatment types were administered from May 2009 to December 2012 and from January 2013 to April 2015, respectively. The inclusion criteria were: 1) symptomatic eyes with treatment-naïve PCV involving the macular area; and 2) best-corrected visual acuity (BCVA) equal to or less than 0.8 in the decimal visual acuity system, using the Landolt chart. Exclusion criteria were: 1) previous history of ocular treatments other than cataract surgery; or 2) other neovascular maculopathy, including typical neovascular AMD, retinal angiomatous proliferation, and choroidal neovascularization secondary to angioid streaks, high myopia, and uveitis. Written informed consent for treatment was obtained from each patient prior to treatment. This retrospective study was approved by the institutional review board of the University of Yamanashi and followed the tenets of the Declaration of Helsinki.

Prior to treatment, all patients underwent a comprehensive ophthalmic examination, including BCVA and intraocular pressure measurements, slit-lamp biomicroscopy using 78D contact lenses, spectral-domain optical coherence tomography (SD-OCT) imaging using the Cirrus OCT system or Spectralis OCT/HRA (Heidelberg Engineering, Dossenheim, Germany), fundus color photography, fluorescein angiography (FA), and indocyanine green angiography (ICGA) using Imagenet2000 (Topcon, Tokyo, Japan) or Spectralis OCT/HRA. All eyes with PCV exhibited characteristic polypoidal dilation with or without branching vascular networks on ICGA.

### Treatment and follow-up

All eyes were treated initially with IVR or IVA, followed by PDT after a one-week interval. The PDT spot size was determined by adding 1000 μm to the greatest linear dimension (GLD) covering the polypoidal lesions and branching vascular networks on ICGA. PDT was performed by one of the authors (Y.S.) according to the standard protocol^36^.

At every follow-up visit, BCVA and intraocular pressure measurements, slit-lamp biomicroscopy, and SD-OCT examinations were routinely performed. After the initial treatment with PDT/IVR or PDT/IVA, patients were followed up every three months until they required retreatment for recurrent exudative changes, which included subretinal or intraretinal fluid, detected by SD-OCT, and a new hemorrhage in the subretinal space or beneath the retinal pigment epithelium, observed with an ophthalmoscope. FA and ICGA were performed on all eyes requiring retreatment. When polypoidal lesions were detected on ICGA, the initial treatment was repeated, and followed up every three months. In the case of recurrence without evidence of polypoidal lesions, additional IVR or IVA as per the initial treatment was repeated monthly without PDT, until the exudative changes disappeared, and thereafter patients were followed up monthly.

### Genotyping

Genotyping of *ARMS2* A69S (rs10490924) and *CFH* I62V (rs800292), which are the two major genetic variants susceptible to AMD in the Japanese population, was performed for all patients. Genomic DNA was extracted from peripheral blood samples and purified using a Puregene DNA Isolation Kit (Gentra Systems, Minneapolis, MN, USA). Genotyping was performed using TaqMan genotyping assays with a 7300/7500 Real-Time PCR System (Applied Biosystems, Foster City, CA, USA) in accordance with the manufacturer’s recommendations, as recently described^37^.

### Statistical analysis

Statistical analysis was performed using DR. SPSS for Window (IBM, Tokyo, Japan).

BCVA measured with the decimal unit system using the Landolt chart was converted to the logarithm of minimum angle resolution (logMAR) for statistical analysis. Differences in categorical variables were tested using the chi-square test. Differences in continuous variables between the two groups were tested using the Mann-Whitney U test. The paired t-test was used to compare the variables before and after treatment. Multivariate linear regression analysis was performed to investigate the baseline factors associated with BCVA improvement at 24 months. Multivariate logistic regression analysis was performed to investigate the baseline risk factors for retreatment due to residual or recurrent exudation. Kaplan-Meier survival analysis and Cox proportional hazards regression analysis were conducted to estimate the risk factors for retreatment. A P-value less than 0.05 was considered statistically significant.
